# The Silicate Cements*Read before the Illinois State Dental Society, May, 1910.

**Published:** 1910-09-15

**Authors:** Geo. C. Poundstone

**Affiliations:** Chicago, Ill.


### THE SILICATE CEMENTS.*

BY GEO. C. POUNDSTONE, D D.S., CHICAGO, ILL.

I was an enthusiast and have used Ascher's Enamel extensively, so the results set forth in this paper will be based upon that product, which is probably at least the equal of any of the silicite cements offered to the dentist today. The large number of fillings inserted, the varying conditions under which they were placed in the teeth and the careful application to detail in manipulation will I trust protect me from

*Read before the Illinois State Dental Society, May, 1910.

the charge of condemning a product without good and sufficient reasons for all of the unfavorable statements that I may make. When I say that I am not an enthusiast I do not mean that I do not use artificial enamel, for I do use it in certain selected cases, but not to the extent that I did, nor do I use it as a permanant filling material. I have fillings of Ascher's Artificial Enamel in the teeth of some patients that were inserted three year ago and they are in apparently as good condition today as when first put in, but to offset this I have replaced a large number of fillings that were inserted with the greatest care and attention to detail in manipulation because they failed in one or more of the points to be spoken of later. I do not believe that all of these failures can be laid to faulty manipulation because if that be true and success will not follow the care and painstaking efforts that I have expended upon it then it will never be a success with the general practitioner, nor can it be true that the conditions existing in certain mouths are entirely responsible for its success or failure because some of my greatest successes stand alongside of the ruins of my greatest failures, and in each of these instances the same amount of care was taken both in the mixing and in the insertion of the material into the cavity.

The failures in these cases have been either a granular surface after a few months with brittle, fractured margins, a discolored filling which may be either slight upon the outer surface or may penetrate the entire mass, a heavy black line around the margin or extending into the space between the filling and the cavity wall and showing through the enamel, disintegration to such an extent that the filling dropped out of the cavity, or the death of the pulp under the filling.

In regard to the manipulation of Ascher's Artificial Enamel I wish to state that the directions of the manufacturers and the demonstrators were followed as closely as possible and after each filling a detailed account of all conditions that might have an influence upon it were written down, the temperature of the room, apparent humidity, time of mixing, condition of the mass when inserted, rapidity of setting, instruments used in inserting and polishing and after comparing and studying this data for months slight advantage was gained because with conditions apparently the same one filling would stand while others would fail.

It is claimed by the manufacturers that artificial enamel is a scientific product, an exact chemical compound. If this be true it can not readily be a success with the dentist unless he also be a chemist. The general practitioner has but a limited knowledge of chemistry, extending no further than that acquired in the regular high school and dental college courses, while the practical, advanced chemical knowledge has been acquired by the few at such a cost in money, time and labor that its secrets have been carefully guarded. The manufacturer prepares a product in two parts which he sells to the dentist, who accepts it with little or no knowledge of what it contains and forwith attempts to complete the chemical problem. If he succeeds it is an accident. If he fails it is because he does not fully comprehend all the details in the process he is trying to bring to a conclusion. If all the chemical combinations were in one part and the other part a simple element as in amalgam, then the dentist might combine the two parts according to a set of directions and secure uniform results, but in cements and enamel both parts are complex combinations in themselves and their union is dependent upon so many conditions as to temperature, humidity, method of mixing and manipulation that accurate results are not readily obtained.

If the manufacturer would take more pains to try to enlighten the dentist as to the chemistry of his product, how and why it combines as it does, and less time to instruction in the mechanical mixing, the dentist would be gaining knowledge from which both he and the manufacturer would profit.

I do not wish to be understood as condemning Artificial Enamel as a filling material, the beauty of which when properly placed can only be approached by the best porcelain inlay, but I do contend that ninety percent. of the dentists before me are having indifferent success; with from fifty to ninety per cent. of the fillings inserted failing within three years and those fillings that were perfect after that time were accidental. That is, an exact chemical compound had been made and the dentist at the time knew not that he had made it. Such uncertainty is not conducive to the establishment of the public confidence in dentists and dentistry that we hope for, and while it may be a little easier money for the dentist at first, every failure will return to him as a boomerang. Artificial enamel should be used only in carefully selected cases, in mouths that are habitually clean, mouths in which the teeth are well shaped, secretions normal, no tendency to the formation of tartar, etc. It is in such mouths that the successful fillings I have made are all to be found and while all the fillings in such mouths have not been successful I think I may safely say that all of the fillings in carelessly kept, unclean mouths have failed or are deteriorating. This would seem to indicate that decomposing food particles in contact with the filling have an effect upon it and may be the reason why so many fillings become dark gray or slate color after a time. At first this gray or slate color can be removed by polishing, but in time it will penetrate the entire filling. Enamel fillings are contraindicated in mouths in which the saliva is large in amount, thick and and viscid, because such mouths can rarely be kept clean for any length of time. From my observations, practically all fillings in such mouths are discolored. Fillings that show a heavy black line around the margin are invariably leaking. This may be caused first by a contraction of the material in setting or it may be that the material was not forced into perfect contact with the cavity wall, sometimes a difficult task, especially if one trys to push the material ahead of an instrument against a wall. It will usually draw away from the wall upon the removal of the instrument. A better way is to draw the plastic material against the wall with a wiping motion whenever possible, thereby tightly sealing the joint between material and cavity wall, not only at the outer margin but over the entire surface of the wall. This tendency to bridge over a space, especially over under cuts, is the cause of many of the black lines around the fillings, for a space cannot exist within a tooth without becoming filled with moisture from the tooth itself. Later this moisture filled space is sure to become discolored and show through the enamel.

Fillings that are discolored throughout the mass are porous to such an extent that moisture and debris can readily penetrate them. Why this is true is difficult to definitely determine, because with the same apparent degree of care in mixing and polishing some fillings will stand impervious while others will become quickly discolored. The demonstrators for the manufacturers will tell you that you used steel instruments, or used vaseline, or didn't wait fifteen minutes, or used a slab that had previously been used for oxyphosphate cement, but when all of these things have been carefully avoided still the same fault will be found in many fillings. Evidently there has not been a perfect chemical compound formed; consequently some element has not been satisfied and is soon washed out or disintegrated, leaving open the way for moisture and debris to enter.

The highly polished surface seems to be the ideal one but in some fillings it is impossible to get such a surface while in others it can be readily produced. The gelatin matrix, if it could be so applied as to make subsequent polishing unnecessary, would possibly give a surface that would remain impervious to moisture, but how can a matrix be so applied? The instant a polishing strip is drawn over the filling all the high gloss left by the gelatin is destroyed and conditions are the same as if no matrix had been used. The matrix can do no more than prevent a large excess of material at inaccessible places, and whether it be gelatin or celluloid can make no difference. The filling must afterwards be polished any way, the same as an amalgam or a gold filling made with a matrix.

The low degree of edge strength is not a serious objection to enamel as a filling material, because there are so many cavities in which the margins are well protected that it can be used in such cases without danger to the margins were that the only difficulty. Careful judgment in selecting cases will obviate fractured margins.

It seems to be a pretty well established fact that pulps die more readily under enamel fillings than under gold or cement fillings. Whether this be due to some chemical poison in the material, I am unable to say, but be it as it may all such danger can be avoided by lining deep cavities in vital teeth with oxyphosphate cement.

I have painted a rather gloomy picture of this most beautiful of all fillings, but there is no use in trying to deceive ourselves. It is a difficult material to work, as difficult as was the porcelain inlay. I do not believe that we have a perfect product as yet, but I have high hopes that a product can be made and will be made that can be worked by the general practitioner of dentistry who has neither the time nor the opportunity to acquire an advanced chemical education.

Until such a product is placed in our hands my warning is to go slow, use the utmost care and judgment in the handling of the material and the selection of the cases in which you use it. Study carefully all the conditions in each case, be extremely careful as to detail in the mixing and insertion into the cavity, and then study the the results in months and years, and thereby try to determine the causes in those cases that fail.

Do not discontinue its use if you have had many failures but be more conservative and thoughtful, and the day may soon come when your conservatism and thoughtfulness will develop a product that will take first rank not only for beauty but for durability and permanence as well.—*Dental Review*, August, 1910.

